# A case–control study of sporadic retinoblastoma in relation to maternal health conditions and reproductive factors: a report from the Children’s Oncology group

**DOI:** 10.1186/s12885-015-1773-0

**Published:** 2015-10-19

**Authors:** Julia E. Heck, Negar Omidakhsh, Saeedeh Azary, Beate Ritz, Ondine S. von Ehrenstein, Greta R. Bunin, Arupa Ganguly

**Affiliations:** 1 Department of Epidemiology, Fielding School of Public Health, University of California, 650 Charles E Young Dr, Box 951772, Los Angeles, CA 90095-1772 USA; 2 Department of Community Health Sciences, Fielding School of Public Health, University of California, 650 Charles E Young Dr, Box 951772, Los Angeles, CA 90095-1772 USA; 3 Division of Oncology and Center for Childhood Cancer Research, Children’s Hospital of Philadelphia, 3535 Market Street, Room 1472, Philadelphia, PA 19104 USA; 4 Department of Genetics, University of Pennsylvania, USA415 Anatomy Chemistry Building, 3620 Hamilton Walk, Philadelphia, PA 19104 USA

**Keywords:** Retinoblastoma, Body mass index, Diabetes, Contraception, Condoms, Human papillomavirus, Fertility Agents, Acetaminophen, Risk Factors, Childhood Cancer Epidemiology

## Abstract

**Background:**

The early age at retinoblastoma occurrence, the most common eye malignancy in childhood, suggests that perinatal factors may contribute to its etiology.

**Methods:**

In a large multicenter study of non-familial retinoblastoma, we conducted structured interviews with the parents of 280 cases and 146 controls to elicit information on health during the perinatal period. We used unconditional logistic regression to assess associations between retinoblastoma and parental fertility treatment, birth control use in the year prior to pregnancy, maternal health conditions and the use of prescription medications during pregnancy, and whether mothers breastfed the index child.

**Results:**

Bilateral retinoblastoma was related to maternal underweight (body mass index <18.5) prior to pregnancy [Odds Ratio (OR) = 4.5, 95 % confidence interval (CI) 1.0, 20]. With regards to unilateral retinoblastoma, we observed a negative association with the use of condoms in the year prior to pregnancy (OR = 0.4, CI 0.2, 0.9), and a trend towards a positive association with maternal diabetes (OR = 2.2, CI 0.8, 6.6).

**Conclusions:**

Results from our study suggest a role for several maternal health and reproductive factors. Given that there are few epidemiologic studies of retinoblastoma, our results require replication in studies which utilize medical record review.

**Electronic supplementary material:**

The online version of this article (doi:10.1186/s12885-015-1773-0) contains supplementary material, which is available to authorized users.

## Background

Retinoblastoma is the most common intraocular malignancy in childhood, with an incidence of 11.8 per million children aged 0–4 years in the United States [1]. It results from inactivation of both alleles of the tumor suppressor *RB1* gene. When the disease is familial (6–10 % of cases [2]) children inherit one *RB1* gene mutation from a parent, and the other allele is lost somatically. The remaining cases of disease are considered sporadic. In 30 % of cases, the *RB1* mutation in one allele occurs as a *de novo* mutation in parental germline cells (usually of paternal origin [3]) or happens in very early embryonic development, and the other allele is lost somatically sometime after conception. In both of the above instances, retinoblastoma typically presents bilaterally. In the remaining 60 % of cases, the disease results from two somatic alterations in a retinal cell at some point after conception; these cases present as unilateral disease.

Although little is known about the causes of sporadic retinoblastoma, there have been several studies that suggested a role for reproductive health factors. A meta-analysis of four studies reported an increased risk for retinoblastoma among children conceived via assisted reproductive technologies [4]; however, a recent study did not support this association [5]. Some, but not all, epidemiologic and laboratory studies have suggested a role for human papillomavirus (HPV) in retinoblastoma [6–15]. A study in Sweden found high but imprecisely estimated increased risks of retinoblastoma with longer breastfeeding, although a dose–response effect was not evident [16].

Parental prescription drug use in pregnancy has been of particular interest in cancer research ever since Herbst and Ulfelder reported an association between maternal use of diethylstilbestrol (DES) in pregnancy and clear cell adenocarcinoma of the vagina in young women [17]. A number of childhood cancer studies have examined the risk from maternal intake of medication during pregnancy [18–20], however studies of retinoblastoma are limited. While one study observed the prevalence of morning sickness to be the same among retinoblastoma cases and controls [21], the use of antinauseants was much higher among case mothers in a different study [7].

The goal of the present study was to examine maternal comorbidities, medication use during pregnancy, and reproductive health related factors in relation to retinoblastoma in offspring.

## Methods

We conducted a multi-center case–control study of retinoblastoma across the USA and Canada from June 2006 to June 2011 [22]. Children with sporadic retinoblastoma were identified from Wills Eye Hospital in Philadelphia, or by the Children’s Oncology Group (COG), which includes over 200 institutions in the USA and Canada. Each participating COG institution, Wills Eye Institute, the University of Pennsylvania, and the University of California, Los Angeles approved the study. After a physician gave his or her approval to contact a patient, children were eligible for the present study if at least one biological parent consented to participate, if they resided in the continental U.S., Alaska, or Canada, if they had a telephone in their household, and if at least one parent spoke English or Spanish. Children conceived with a donor egg or sperm were eligible for the study. Participants provided written informed consent for biospecimen collection; because interviews took place via telephone, we collected verbal informed consent for the interview, which was documented on the questionnaire. In total, 282 cases of retinoblastoma (187 unilateral and 95 bilateral cases) were enrolled in the study.

The family of each case was asked to nominate one or more children of their friends or non-blood relatives under age 15 as potential controls. For bilateral cases, eligible control fathers were not a biological relative of the case’s father; for unilateral cases, eligible control mothers were not a biological relative of the case’s mother. We attempted to match controls to cases on the child’s age at the time of interview (0–1, 2–4, 5–9, 10–14 years old). If more than one control was nominated by the case parents, we attempted to recruit the eligible control closest in age to the case. If that control was not successfully recruited, we attempted to recruit the next control until we obtained a control or until we contacted all potential controls. When an “ideal control” (age-matched and not a biological relative) was unavailable, cases were asked to suggest another potential child as a control. Some families did not nominate any child as a control or the control’s family did not consent to enroll in the study, therefore for some cases there was no matched control. Overall, 155 controls were recruited.

The majority (>90 %) of sporadic bilateral cases are due to a *de novo* mutation in the father’s germline [3]. Therefore, for bilateral cases, we were most interested in paternal preconceptional exposures. In contrast, because unilateral cases derive from two *RB1* mutations which occur during pregnancy, for unilateral cases we were most interested in examining pregnancy exposures. In a structured telephone interview with the parents, data were collected on demographic information, the mother’s medical conditions in pregnancy, her reproductive history, and other exposures. With regards to perinatal health conditions, mothers were asked several open-ended questions to prompt their memories about pregnancy-related and unrelated medical conditions which occurred in the month prior or during pregnancy, as well as conditions that had been diagnosed before the pregnancy but for which they had received treatment during the pregnancy. With regards to medications, mothers were asked “Did you…take prescription medicine for any condition, such as the flu, an infection, accident or injury, in the month before or during your pregnancy?” Over-the-counter medications were not ascertained. In total, 280 mothers of cases (185 unilateral and 95 bilateral) and 146 mothers of controls completed the interview. When one parent was unavailable, the interview was conducted with a proxy who was typically the other parent, with 16.5 % of paternal and 3 % of maternal interviews conducted by proxy.

We used unconditional logistic regression to evaluate the risk of retinoblastoma. Given that a number of cases had no matched control, we chose to use unconditional logistic regression in order to improve statistical power. We reported odds ratios (ORs) and 95 % confidence intervals (CIs) adjusted for mother’s race/ethnicity (White non-Hispanic, Black non-Hispanic, Hispanic, other), maternal educational attainment (Less than high school, high school graduate, some college or other training, college graduate or more), household income (less than $35,000, $35,000 to 50,000, $50,000 to 75,000, more than $75,000), the mother’s age at child’s birth (continuous), and a behavioral indicator, the mother’s tobacco smoking in the month before or during pregnancy (Yes/No). We explored additional adjustment for marital status and child’s gender however they did not change the estimates by more than 10 %, and were not included in the final model. When statistical power allowed us to do so, we checked our results in the matched analysis using conditional logistic regression, adjusting for the same covariates other than child’s age (matching variable).

Many health conditions were identified by only a small number of mothers. We provide results for which there were at least 5 unilateral cases that reported having the medical condition; in addition, because of the prior reported associations between retinoblastoma and infertility treatment [4] as well as sexually transmitted diseases [6], we reported associations with any sexually transmitted disease and with the type of fertility treatment. The category “other viral infections” included hepatitis B and C, shingles, HPV, herpes, stomach virus, Murray infection, and Fifth disease. We defined hormonal birth control methods as oral contraceptive pills, injection, implant, skin patch, or vaginal ring. The category “other types of birth control” included diaphragm, cervical cap, sponge, IUD, Lea’s shield, other barrier method, vasectomy, tubal ligation, rhythm method, fertility awareness, and withdrawal.

We examined maternal and paternal weight prior to pregnancy and pregnancy weight gain. Based upon recommendations issued by the Institute of Medicine [23], we defined normal weight gain in pregnancy as 28–40 pounds for underweight women [body mass index (BMI) < 18.5], 25–35 pounds for normal women (BMI = 18.5–25), 15–25 pounds for overweight women (BMI = 25–30) and 11–20 pounds for obese women (BMI > 30).

In models which evaluated retinoblastoma’s association with birth control use, we adjusted for the same variables above except the mother’s age at child’s birth, because it did not change the point estimate by more than 10 %. For analyses of fertility treatment, parity and breast feeding, we utilized the same covariates in models except for mother’s age at child’s birth and mother’s tobacco smoking, because they did not change point estimates by more than 10 %.

Because information for some covariates was missing (primarily with regards to family income and maternal smoking status during pregnancy) we conducted sensitivity analyses in which we used multiple imputation methods (“PROC MI and PROC MIANALYZE”) in SAS 9.2 to compensate for missing data. In addition, due to differences between case and control groups, we then analyzed our imputed dataset using propensity score techniques where scores for all exposures were calculated from a logistic regression model with each exposure from tables 2, 3 and 4 set as the dependent variable and all covariates set as the independent variable. Results from the multiple imputation/propensity score analyses did not differ substantially from the main results, with most point estimates and confidence intervals changing by <0.1–0.2. Thus, we present results from logistic regression in main tables; multiple imputation/propensity score analyses are presented in Additional file 1.

We additionally conducted sensitivity analyses to examine whether results changed when we excluded the 3 % of maternal interviews that were conducted by proxy.

## Results

On average, bilateral cases were diagnosed at age 1.1 years [standard deviation (sd) = 0.9] and unilateral cases were diagnosed at age 2.0 years (sd = 1.7). Table 1 shows the demographic characteristics of participants. Mothers of unilateral cases included more Hispanics or Blacks and fewer whites than mothers of controls. Mothers of bilateral and unilateral cases had lower educational attainment and income than control mothers. There was also some indication that mothers of bilateral cases were older (35+ years old) during pregnancy than control mothers. Moreover, mothers of both bilateral and unilateral cases were more likely to smoke in the month before or during pregnancy.

**Table 1:** Demographic characteristics of mothers

	Controls	Unilateral cases	Bilateral cases
Characteristics	(*N* = 146)	(*N* = 185)	(*N* = 95)
	N (%)	N (%)	N (%)
Mother’s race			
White non-Hispanic	111 (76)	105 (56.8)	66 (69.5)
Black non-Hispanic	5 (3.4)	14 (7.6)	3 (3.2)
Hispanic	18 (12.3)	46 (24.9)	12 (12.6)
Other	12 (8.2)	20 (10.8)	14 (14.7)
Mother’s educational attainment			
Less than High school	6 (4.1)	15 (8.1)	9 (9.5)
High school graduate	15 (10.3)	32 (17.3)	13 (13.7)
Some college or other training	26 (17.8)	40 (21.6)	16 (16.8)
College graduate or more	99 (67.8)	98 (53.0)	57 (60.0)
Mother’s age at child’s birth			
< 25	20 (13.7)	31 (16.8)	17 (17.9)
25–29	45 (30.8)	59 (31.9)	25 (26.3)
30–34	58 (39.7)	61 (33.0)	29 (30.5)
35–39	21 (14.4)	26 (14.1)	21 (22.1)
40+	2 (1.4)	8 (4.3)	3 (3.2)
Total household income			
Less than $35,000	29 (19.9)	56 (30.3)	27 (28.4)
$35,000–50,000	19 (13.0)	18 (9.7)	11 (11.6)
$50,000–75,000	30 (20.6)	30 (16.2)	18 (19.0)
More than $75,000	60 (41.1)	62 (33.5)	32 (33.7)
Refused/do not know	8 (5.5)	19 (10.3)	7 (7.4)
Mother smoked in the month before or during pregnancy			
Yes	17 (11.6)	42 (22.7)	19 (20.0)
No	127 (87.0)	142 (77.2)	75 (78.9)
Missing	2 (1.4)	1 (76.8)	1 (1.1)

Associations between maternal medical problems in the month before or during pregnancy and sporadic retinoblastoma development in their child are presented in Table 2; only a few mothers reported any of a number of conditions during pregnancy. Few parents reported any sexually transmitted disease in pregnancy; reported infections included one case of HPV and one of herpes. Reports of diabetes also showed increases in unilateral and bilateral retinoblastoma (OR = 2.2, CI 0.8, 6.6, and OR = 1.9, CI 0.6, 6.6, respectively). Despite the low number of exposed controls (*N* = 1), we did find a trend towards increased risk of unilateral retinoblastoma among mothers with anemia (OR = 2.8, CI 0.3, 27.0). Use of prescription pain medication in pregnancy was positively associated with unilateral and bilateral retinoblastoma development in the child (OR = 9.0, CI 1.4, 56.4), however this association was tempered in multiple imputation/propensity score analyses (OR = 3.7, CI 0.9, 16.1). In conditional logistic regression analyses, however, no association was observed for either unilateral (OR = 0.9, CI 0.03, 26.3) nor bilateral (OR = 1.2, CI 0.1, 25.8) disease. Of the pain medications used, 12 mothers took acetaminophen, 3 took ibuprofen, 1 took tramadol, and the remaining 2 took an unspecified type of medication. Mothers of both unilateral and bilateral cases reported more diabetes (OR = 2.2, CI 0.8, 6.6 using unconditional logistic regression; OR = 2.3, 95 % CI 0.9, 6.0 in multiple imputation/propensity score analyses). Mothers of cases also reported more depression or anxiety disorders (OR = 3.3, CI 0.7, 14.8, OR = 3.8, CI 0.7, 21.8) and asthma/allergies (bilateral) (OR = 1.7, CI 0.5, 5.9) during their pregnancy with their child than control mothers.

**Table 2:** Retinoblastoma in relation to maternal medical conditions and prescription drug use

	Controls (*n* = 136)	Unilateral cases (*n* = 165)			Bilateral cases (*n* = 87)		
			Crude^a^	Adjusted^b^		Crude^a^	Adjusted^b^
*Medical conditions*	N (%)	N (%)	OR	OR (95 % CI)	N (%)	OR	OR (95 % CI)
Any medical condition	74 (47.7)	115 (61.5)	1.6	1.5 (0.9, 2.6)	57 (60.0)	1.6	1.2 (0.7, 2.3)
Infectious diseases							
Any infectious disease	32 (20.7)	44 (23.6)	1.1	1.0 (0.5, 1.7)	19 (20.0)	0.8	0.8 (0.4, 1.6)
Respiratory infection	18 (11.6)	21 (11.2)	1.0	0.9 (0.4, 2.0)	7 (7.4)	0.5	0.4 (0.1, 1.2)
Flu or cold	10 (6.5)	17 (9.1)	1.4	1.2 (0.5, 3.1)	3 (3.4)	0.4	0.3 (0.1, 1.4)
Other viral infections	1 (0.7)	5 (2.7)	3.8	3.6 (0.3, 47.9)	2 (2.1)	2.2	3.7 (0.3, 3.5)
Sexually transmitted infections	0 (0.0)	1 (0.5)	-	-	1 (1.1)	-	-
All bacterial infections	18 (11.6)	24 (12.8)	1.1	1.1 (0.5, 2.4)	11 (11.6)	0.9	0.8 (0.3, 2.1)
Urinary tract infections	6 (3.9)	9 (4.8)	1.1	0.8 (0.2, 3.0)	1 (1.1)	0.1	0.2 (0.1, 1.8)
Mother took antibiotics in pregnancy	21 (13.6)	22 (11.8)	0.8	0.8 (0.3, 1.8)	10 (10.5)	0.6	0.5 (0.2, 1.4)
Chronic diseases							
High blood pressure	17 (11.0)	23 (12.3)	1.1	1.2 (0.5, 2.6)	11 (11.6)	1.2	1.0 (0.4, 2.6)
Gestational high blood pressure	17 (11.0)	22 (11.8)	1.1	1.1 (0.5, 2.5)	11 (11.6)	1.2	1.0 (0.4, 2.6)
Diabetes (any)	6 (3.9)	17 (9.1)	2.5	2.2 (0.8, 6.6)	8 (8.4)	1.8	1.9 (0.6, 6.6)
Gestational diabetes	6 (3.9)	14 (7.5)	2.0	1.9 (0.6. 5.7)	8 (8.4)	1.8	1.9 (0.6, 6.6)
Preeclampsia	7 (4.5)	9 (4.8)	1.1	1.1 (0.3, 4.4)	2 (2.1)	0.7	0.6 (0.1, 4.0)
Endocrine disorders							
Any thyroid problem	2 (1.3)	6 (3.2)	2.2	2.2 (0.4, 12.3)	2 (2.1)	1.0	0.8 (0.1, 7.5)
Hypothyroidism	2 (1.3)	5 (2.7)	1.6	1.6 (0.3, 9.6)	2 (2.1)	1.0	0.8 (0.1, 7.4)
Pain and pain medication							
Back pain	4 (2.6)	11 (5.9)	2.5	3.4 (0.8, 14.4)	4 (4.2)	2.2	2.0 (0.3, 13.9)
Pain medication	3 (1.9)	8 (4.3)	2.3	4.2 (0.8, 23.3)	7 (7.4)	6.0	9.0 (1.4, 56.4)
Other Diseases and conditions							
Depression/anxiety	5 (3.2)	10 (5.4)	2.2	3.3 (0.7, 14.8)	5 (5.3)	2.3	3.8 (0.7, 21.8)
Anemia	1 (0.7)	6 (3.2)	4.5	2.8 (0.3, 27.0)	2 (2.1)	2.2	1.1 (0.1, 16.1)
Allergy/asthma	8 (5.2)	13 (7.0)	1.3	1.3 (0.5, 3.7)	7 (7.4)	1.3	1.7 (0.5, 5.9)

^a^Adjusted for the matching variable, child’s age at interview

^b^Adjusted for child age at interview, mother’s race/ethnicity, mother’s educational attainment, household income, mother’s age at birth, and maternal smoking in the month before or during pregnancy

Maternal underweight prior to pregnancy was associated with an increase in bilateral retinoblastoma (Table 3), although results were lower in multiple imputation/propensity score analyses. In the conditional analyses, maternal underweight was also positively associated with unilateral retinoblastoma (OR = 4.0, 95 % CI 0.5, 31.5), while the association with bilateral disease was not possible to estimate because there were no underweight case mothers. We observed that breast feeding decreased the risk of unilateral retinoblastoma, and this protective effect was stronger for children who had been breast fed for 7–11 months. However, no dose–response effect was observed with longer (12+ months) breastfeeding. When we stratified by maternal education, we observed that longer breastfeeding was negatively associated with retinoblastoma for mothers with a high school diploma or fewer years of education (OR = 0.2, CI 0.01, 1.6) and among mothers who had some schooling after high school but who had not graduated college (OR = 0.2, CI 0.008, 3.0). In contrast with the mothers with less formal education, breastfeeding was associated with increased risk among women with a college degree or more (OR = 2.6, CI 0.9, 8.0). In conditional logistic regression analyses, breastfeeding greater than 12 months was associated with a weak decreased risk (OR = 0.5, CI 0.1, 2.1).

**Table 3:** Associations between maternal pregnancy history, body size, and breastfeeding with retinoblastoma using unconditional logistic regression

	Controls	Unilateral cases (*n* = 165)			Bilateral cases (*n* = 87)		
	(*N* = 136)		Crude^a^	Adjusted^b^		Crude^a^	Adjusted^b^
	N (%)	N (%)	OR	OR (95 % CI)	N (%)	OR	OR (95 % CI)
Maternal history of stillbirth	1 (0.7)	5 (2.7)	4.8	2.1 (0.2, 20.9)	2 (2.1)	4.1	2.9 (0.2, 49.8)
Order of index pregnancy							
First pregnancy	55 (38.2)	60 (32.6)	1.0	Referent	33 (35.1)	1.0	Referent
Second pregnancy	39 (27.1)	57 (31.0)	1.4	1.4 (0.8, 2.7)	32 (34.0)	1.6	1.8 (0.8, 3.8)
Third and above	50 (34.7)	67 (36.4)	1.3	1.2 (0.7, 2.2)	29 (30.9)	1.1	1.2 (0.6, 2.4)
Order of index child							
First child	71 (49.3)	86 (46.7)	1.0	Referent	46 (49.5)	1.0	Referent
Second child	40 (27.8)	68 (37.0)	1.5	1.6 (0.9, 2.8)	27 (29.0)	1.2	1.3 (0.6, 2.7)
Third and above	33 (22.9)	30 (16.3)	0.8	0.7 (0.4, 1.4)	20 (21.5)	1.1	1.1 (0.5, 2.5)
Ever breastfed index child	120 (85.1)	137 (74.9)	0.5	0.5 (0.2, 0.9)	74 (79.6)	0.6	0.7 (0.3, 1.6)
Breastfeeding duration							
None or <1 month	25 (18.7)	47 (28.5)	1.0	Referent	19 (24.4)	1.0	Referent
1–6 months	66 (49.3)	67 (40.6)	0.5	0.5 (0.3, 0.9)	36 (46.2)	0.7	0.8 (0.3, 1.8)
7–11 months	21 (15.7)	16 (9.7)	0.4	0.3 (0.1, 0.8)	7 (9.0)	0.4	0.6 (0.2, 1.8)
12+ months	22 (16.4)	35 (21.2)	0.8	1.0 (0.4, 2.2)	16 (20.5)	1.1	1.0 (0.3, 2.7)
Gave index child formula while breastfeeding	68 (58.1)	78 (56.9)	1.0	0.9 (0.5, 1.5)	38 (54.3)	0.8	0.7 (0.4, 1.5)
Mother’s BMI at the start of pregnancy							
Underweight (BMI:<18.5)	5 (3.5)	10 (5.4)	1.9	2.6 (0.7, 9.4)	8 (8.4)	3.8	4.5 (1.0, 20.1)
Normal (BMI: 18.5- < 25)	90 (62.1)	101 (54.9)	1.0	Referent	50 (52.6)	1.0	Referent
Overweight (BMI:25- < 30)	31 (21.4)	45 (24.5)	1.3	1.2 (0.7, 2.3)	20 (21.1)	1.3	1.2 (0.5, 2.6)
Obese (BMI: ≥ 30)	19 (13.1)	28 (15.2)	1.4	1.1 (0.5, 2.5)	17 (17.9)	1.6	1.0 (0.4, 2.5)
Weight gain in pregnancy							
Low weight gain	23 (17.0)	26 (14.7)	0.9	0.8 (0.4, 1.8)	12 (13.0)	0.8	0.8 (0.3, 2.0)
Normal weight gain	51 (37.8)	64 (36.2)	1.0	Referent	34 (37.0)	1.0	Referent
High weight gain	61 (45.2)	87 (49.2)	1.1	1.1 (0.6, 1.8)	46 (50.0)	1.0	0.8 (0.4, 1.6)
Father’s BMI at the start of pregnancy							
Underweight (BMI:<18.5)	1 (0.7)	2 (1.2)	2.8	2.2 (0.1,34.6)	2 (2.2)	3.2	3.1 (0.2, 47.5)
Normal (BMI: 18.5- < 25)	36 (25.7)	33 (20.1)	1.0	Referent	32 (35.2)	1.0	Referent
Overweight (BMI:25- < 30)	57 (40.7)	84 (51.2)	1.6	1.7 (0.9, 3.1)	33 (36.3)	0.7	0.7 (0.4, 1.5)
Obese (BMI: ≥ 30)	76 (32.8)	45 (27.4)	1.1	1.0 (0.4, 1.9)	24 (25.4)	0.6	0.6 (0.3, 1.3)

^a^ Adjusted for the matching variable, child’s age at interview

^b^Adjusted for the child’s age at interview, mother’s race/ethnicity, mother’s educational attainment, and household income

The use of condoms for birth control prior to pregnancy was associated with a lower risk of unilateral retinoblastoma in unconditional (OR, 0.4; CI 0.2–0.9; Table 4) and conditional analyses (OR = 0.1, CI 0.1, 0.9). There was a slightly increased risk for unilateral retinoblastoma and the use of any type of hormonal birth control; this estimate did not change after additional adjustment for condom use. Elevated point estimates, with wide confidence intervals, could be seen when examining the association with fertility treatment. In conditional regression analyses, there was no association between the use of any type of fertility treatment and unilateral disease (OR = 0.9), however an increased risk for bilateral disease, with wide confidence intervals, was seen (OR = 2.4, CI 0.1, 20.2). The types of fertility treatment that were reported were In-Vitro Fertilization [IVF; 0 controls, 7 (3.8 %) unilateral cases, 5 (5.3 %) bilateral cases], Intra Cytoplasmic Sperm Injection [ICSI; 1 (0.7 %) controls, 3 (1.6 %) unilateral cases, 2 (2.1 %) bilateral cases], and artificial intra-uterine insemination [2 (1.4 %) controls, 3 (1.6 %) unilateral cases, 3 (3.2 %) bilateral cases]. Of the participants that could not identify the specific type of fertility treatment used, 2 controls, 2 unilateral cases, and 4 bilateral cases indicated it was due to a problem of the mother’s, while no controls, 3 unilateral cases, and 2 bilateral cases indicated it was due to a problem of the father’s. It should be noted that the same families may have tried several different fertility treatment methods.

**Table 4:** Sporadic retinoblastoma in relation to the mother’s birth control use and fertility treatment

	Controls	Unilateral (*n* = 165)			Bilateral (*n* = 87)		
	(*N* = 136)		Crude^a^	Adjusted		Crude^a^	Adjusted
	N (%)	N (%)	OR	OR (95 % CI)	N (%)	OR	OR (95 % CI)
Birth control used in the year before the index pregnancy^b^							
Any birth control	75 (52.5)	85 (46.6)	0.8	0.9 (0.5, 1.5)	45 (47.9)	0.8	0.7 (0.4, 1.4)
Oral contraceptive pills	43 (29.3)	60 (32.4)	1.2	1.5 (0.8, 2.5)	28 (29.5)	0.8	0.8 (0.4, 1.6)
Injection, Implant, skin patch, Vaginal ring	9 (6.1)	13 (7.0)	1.2	1.1 (0.4, 3.3)	7 (7.4)	1.0	0.9 (0.3, 3.5)
Any hormonal contraceptive	49 (33.3)	71 (38.4)	1.3	1.5 (0.9, 2.6)	34 (35.8)	0.9	0.9 (0.5, 1.7)
Condoms	26 (17.7)	15 (8.1)	0.4	0.4 (0.2, 0.9)	14 (14.7)	0.9	0.8 (0.4, 1.9)
Intra uterine device (IUD)	4 (2.7)	3 (1.6)	0.6	0.4 (0.1, 2.1)	1 (1.1)	0.4	0.4 (0.0, 4.8)
Other type of birth control	2 (1.4)	2 (1.1)	0.9	1.2 (0.2, 9.0)	0 (0.0)	-	-
Child conceived with the help of fertility treatment^c^							
Any type of fertility treatment used by either parent	9 (6.3)	14 (7.7)	1.1	1.4 (0.5, 3.5)	8 (8.5)	1.2	1.5 (0.5, 4.5)
Mother took fertility medication for this pregnancy	8 (5.6)	14 (7.6)	1.3	1.6 (0.6, 4.1)	6 (6.4)	1.0	1.2 (0.3, 4.1)
Used IVF for this pregnancy	0 (0)	7 (3.8)	-	-	5 (5.3)	-	-
Used artificial intrauterine insemination	2 (1.4)	3 (1.6)	1.1	1.3 (0.2, 8.3)	3 (3.2)	2.0	2.3 (0.3, 18.0)
Used Intra Cytoplasmic Sperm Injection (ICSI)	1 (0.7)	3 (1.6)	1.9	2.0 (0.2, 23.3)	2 (2.1)	1.9	2.4 (0.2, 28.9)
Problem with egg was the reason for infertility treatment	2 (1.4)	2 (1.1)	0.9	1.1 (0.1, 10.1)	4 (4.3)	2.9	4.0 (0.6, 29.0)
Problem with sperm was the reason for infertility treatment	0 (0.0)	3 (1.6)	-	-	2 (2.1)	-	-
The reason for infertility treatment was unknown	3 (2.1)	7 (3.8)	1.6	2.3 (0.6, 9.4)	2 (2.1)	0.8	1.3 (0.2, 9.1)

^a^ Adjusted for matching variable, child’s age at interview

^b^Adjusted for child’s age at interview, mother’s race/ethnicity, mother educational attainment, household income, and if the mother smoked in the month before and during pregnancy

^c^ Adjusted for child’s age at interview, mother’s race/ethnicity, mother’s educational attainment, and household income

In sensitivity analyses which excluded proxy interviews, we observed similar results to those seen in the overall study.

## Discussion

This study is one of only a small number of investigations into maternal health and reproductive health related factors in relation to retinoblastoma. We observed a negative association for retinoblastoma with condom use in the year before pregnancy and an increased risk with maternal underweight at the start of pregnancy and when the mother took pain medication during pregnancy. For many self-reported health conditions, there were only a small number of mothers who indicated that they had had the condition in the perinatal period, thus we were underpowered in many analyses. Hence, results must be interpreted with caution. Although the small sample size led to wide confidence intervals, we observed trends towards increased risks with several maternal conditions during pregnancy, including viral infections, diabetes, depression or anxiety, and anemia. Other studies examining these conditions have found conflicting results [6, 7]. These findings require replication elsewhere, preferably in studies which utilize medical record review.

Maternal underweight prior to pregnancy is a well-recognized risk factor for a variety of adverse fetal outcomes such as intrauterine growth restriction [24], however there are only a few studies which examined it in relation to childhood cancers. Maternal underweight (BMI <20) was associated with a 40 % increase in hepatoblastoma risk in one population-based study [25]. Maternal undernutrition can alter fetal programming and potentially change specific cell groups or organogenesis including the developing retina [26]. Both animal and human studies have shown that fetal growth restriction can cause changes in retinal structure [27, 28]. A role for specific nutrients in retinoblastoma prevention has been suggested, particularly for folate, an important methyl donor [21, 29].

Our findings on condom use reinforce the observation of an earlier study which reported that the use of barrier conception in the year prior to pregnancy was associated with lower risk of retinoblastoma [7]. Given that the use of condoms not only can prevent incident HPV infections but also may promote clearance of HPV in women with a previously positive HPV test [30, 31], our results support a possible role for HPV in retinoblastoma development. Vertical transmission of HPV would presumably occur during passage of the fetus through the birth canal [32]. In a previous study, we observed a weak lowered risk of unilateral retinoblastoma with Cesarean Section (OR=0.8) [6]. In some but not all studies, HPV DNA has been isolated from retinoblastoma tumor tissue [8–15]. Contradictory findings across studies may be due to variation in the underlying prevalence of HPV across regions, cofactors (dietary or behavioral) that may affect HPV behavior, or specimen contamination. The biological plausibility of this association is supported by the well-known binding of HPV protein E7 to the tumor suppressor protein pRb, rendering it inactive [33].

In the United States, 12 % of women ages 15–44 receive infertility services at some point in their lives, and 1 % of children born each year are conceived through assisted reproductive technologies [34]. Although only a small number of our study participants reported having treatment for infertility, we observed a trend towards increased risk for retinoblastoma with infertility treatment. In our California studies, we observed increased cancer risk among the offspring of multiple births, particularly among embryonal tumors; this may support associations with infertility treatment [6, 35–37]. Although the rarity of childhood cancer has limited research in this area, studies suggest an increased risk for childhood cancers among children conceived after infertility treatments, including a meta-analysis of four studies which observed increases in risk for retinoblastoma (RR = 1.62) [4, 38]. Possible mechanisms that may explain this increased cancer risk include epigenetic changes such as altered DNA methylation and changes in chromatin structure, which may cause imprinting disorders and modified gene expression [39]. However, there is debate as to whether the source of any increased risk for childhood cancer may be the infertility treatment itself or rather due to factors related to the underlying fertility problems.

While breastfeeding confers a number of health benefits to infants, it may also expose the newborn to elevated levels of varying exogenous chemicals that can be found in milk, such as pharmaceuticals taken by the mother, heavy metals, or volatile organic compounds [40]. Our finding of a negative association with breastfeeding was not confirmed in dose–response analyses, which examined duration of breastfeeding. Because we did not collect the reason that parents ceased breastfeeding, it is not known if case parents’ weaning was a result of the retinoblastoma diagnosis.

We additionally observed an increased risk for retinoblastoma with maternal intake of prescription pain medication during pregnancy. The majority of mothers who had taken medication reported intake of acetaminophen (paracetamol). However, misclassification of exposure is a concern given that there were likely a considerable number of mothers in our study who took over-the-counter acetaminophen during their pregnancies. In a pooled analysis of two large studies, 56 % of US women reported taking acetaminophen while pregnant [41]. Despite that acetaminophen is a commonly recommended pain medication for use in pregnancy, there are reports of increased risk of adverse childhood health conditions such as preterm birth, asthma, and hyperkinetic disorders with acetaminophen use [42–44].

A limitation of our study is the possibility of recall errors. In retrospective studies in which mothers are queried about pregnancy health, mothers do best at remembering serious health conditions or major events. For example, hypertension, diabetes, and previous stillbirths tend to be recalled quite accurately, while recall of anemia is more moderate [45, 46]. Mothers tend to remember medications taken for chronic conditions more accurately than those taken for only a short time period [47], and accuracy in recall of short-term medication may differ between cases and controls [48]. However, maternal health conditions were reported in our study at a similar prevalence to rates observed in recent large-scale epidemiologic studies in the US and elsewhere. Pregnancy prevalence of chronic hypertension is 0.5–3 % and gestational hypertension, 4–14 % [49, 50]; chronic diabetes is 1–2 % and gestational diabetes is 8 % [51]; asthma is seen in 4–9 % of US pregnant women [52] and hypothyroidism in 2–3 % [53]. In contrast, the prevalence of depression was much lower in our study than has been seen in other US surveys (10–12 % [54, 55]), potentially due to parental reluctance to disclose mental health problems to interviewers.

Another limitation in this study is the possibility of selection bias in the form of overmatching. Due to the use of friend controls, it is likely that maternal characteristics of cases and controls would be similar. However, differing response rates resulted in controls who tended to be White, more educated, and of higher SES. In matched/conditional analyses, cases without matched controls would drop out of the analysis, while unconditional analyses would need to be controlled for these factors to mitigate the effect of bias due to overmatching. In our analyses, we attempted to control for race, education, and SES, though it may not have completely controlled for all bias due to selection. As a consequence, the forced similarities between cases and controls are likely to bias results to the null.

## Conclusion

In conclusion, we observed retinoblastoma to be associated with several maternal medical conditions and perinatal health-related behaviors. There are few case–control studies on retinoblastoma in the US or internationally, and our results require replication elsewhere, ideally in studies which utilize pharmacy or medical records. Although the public health impact of these associations is likely to be small, our study raises the possibility of several associations, and confirmation of our observations may contribute to prevention recommendations for pregnant women.

## Additional file

Additional file 1: **Table S1.** Multiple imputation/propensity score analysis of retinoblastoma in relation to maternal medical conditions and prescription drug use which occurred in the month before or during the pregnancy. **Table S2.** Multiple imputation/propensity score analysis of associations between maternal pregnancy history, body size, and breastfeeding with retinoblastoma using unconditional logistic regression. **Table S3.** Multiple imputation/propensity score analysis of sporadic retinoblastoma in relation to the mother’s birth control use and fertility treatment. (DOC 141 kb)

## References

[1] Broaddus E, Topham A, Singh AD (2009). Incidence of retinoblastoma in the USA: 1975–2004. Br J Ophthalmol.

[2] Leiderman YI, Kiss S, Mukai S (2007). Molecular genetics of RB1--the retinoblastoma gene. Semin Ophthalmol.

[3] Kato MV, Ishizaki K, Shimizu T, Ejima Y, Tanooka H, Takayama J, Kaneko A, Toguchida J, Sasaki MS (1994). Parental origin of germ-line and somatic mutations in the retinoblastoma gene. Hum Genet.

[4] Hargreave M, Jensen A, Toender A, Andersen KK, Kjaer SK (2013). Fertility treatment and childhood cancer risk: a systematic meta-analysis. Fertil Steril.

[5] Williams CL, Bunch KJ, Stiller CA, Murphy MF, Botting BJ, Wallace WH, Davies M, Sutcliffe AG (2013). Cancer risk among children born after assisted conception. New Engl J Med.

[6] Heck JE, Lombardi CA, Meyers TJ, Cockburn M, Wilhelm M, Ritz B (2012). Perinatal characteristics and retinoblastoma. Cancer Causes Control.

[7] Bunin GR, Meadows AT, Emanuel BS, Buckley JD, Woods WG, Hammond GD (1989). Pre- and postconception factors associated with sporadic heritable and nonheritable retinoblastoma. Cancer Res.

[8] Anand B, Ramesh C, Appaji L, Kumari BS, Shenoy AM, Nanjundappa, Jayshree RS, Kumar RV (2011). Prevalence of high-risk human papillomavirus genotypes in retinoblastoma. Br J Ophthal.

[9] Shetty OA, Naresh KN, Banavali SD, Shet T, Joshi R, Qureshi S, Mulherkar R, Borges A, Desai SB (2012). Evidence for the presence of high risk human papillomavirus in retinoblastoma tissue from nonfamilial retinoblastoma in developing countries. Pediatr Blood Cancer.

[10] Montoya-Fuentes H, de la Paz R-MM, Villar-Calvo V, Suarez-Rincon AE, Ornelas-Aguirre JM, Vazquez-Camacho G, Orbach-Arbouys S, Bravo-Cuellar A, Sanchez-Corona J (2003). Identification of DNA sequences and viral proteins of 6 human papillomavirus types in retinoblastoma tissue. Anticancer Res.

[11] Palazzi MA, Yunes JA, Cardinalli IA, Stangenhaus GP, Brandalise SR, Ferreira SA, Sobrinho JS, Villa LL (2003). Detection of oncogenic human papillomavirus in sporadic retinoblastoma. Acta Ophthalmol Scand.

[12] Orjuela M, Castaneda VP, Ridaura C, Lecona E, Leal C, Abramson DH, Orlow I, Gerald W, Cordon-Cardo C (2000). Presence of human papilloma virus in tumor tissue from children with retinoblastoma: an alternative mechanism for tumor development. Clin Cancer Res.

[13] Bhuvaneswari A, Pallavi VR, Jayshree RS, Kumar RV (2012). Maternal transmission of human papillomavirus in retinoblastoma: A possible route of transfer. Indian J Med Paediatr Oncol.

[14] Ryoo NK, Kim JE, Choung HK, Kim N, Lee MJ, Khwarg SI (2013). Human papilloma virus in retinoblastoma tissues from Korean patients. Korean J Ophthalmol.

[15] Gillison ML, Chen R, Goshu E, Rushlow D, Chen N, Banister C, Creek KE, Gallie BL (2007). Human retinoblastoma is not caused by known pRb-inactivating human DNA tumor viruses. Int J Cancer.

[16] Hardell L, Dreifaldt AC (2001). Breast-feeding duration and the risk of malignant diseases in childhood in Sweden. Eur J Clin Nutr.

[17] Herbst AL, Ulfelder H, Poskanzer DC (1971). Adenocarcinoma of the vagina. Association of maternal stilbestrol therapy with tumor appearance in young women. New Engl J Med.

[18] Cardy AH, Little J, McKean-Cowdin R, Lijinsky W, Choi NW, Cordier S, Filippini G, Holly EA, Lubin F, McCredie M (2006). Maternal medication use and the risk of brain tumors in the offspring: the SEARCH international case–control study. Int J Cancer.

[19] Kwan ML, Metayer C, Crouse V, Buffler PA (2007). Maternal illness and drug/medication use during the period surrounding pregnancy and risk of childhood leukemia among offspring. Am J Epidemiol.

[20] Ross JA, Xie Y, Davies SM, Shu XO, Pendergrass TW, Robison LL (2003). Prescription medication use during pregnancy and risk of infant leukemia (United States). Cancer Causes Control.

[21] Orjuela MA, Titievsky L, Liu X, Ramirez-Ortiz M, Ponce-Castaneda V, Lecona E, Molina E, Beaverson K, Abramson DH, Mueller NE (2005). Fruit and vegetable intake during pregnancy and risk for development of sporadic retinoblastoma. Cancer Epidemiol Biomarkers Prev.

[22] Lombardi C, Ganguly A, Bunin GR, Azary S, Alfonso V, Ritz B, Heck JE (2015). Maternal diet during pregnancy and unilateral retinoblastoma. Cancer Causes Control.

[23] Rasmussen KM, Yaktine AL. Weight gain during pregnancy: reexamining the guidelines. National Academies Press; Washington DC 2009. http://www.ncbi.nlm.nih.gov/books/NBK32813/20669500

[24] Cnattingius S, Bergstrom R, Lipworth L, Kramer MS (1998). Prepregnancy weight and the risk of adverse pregnancy outcomes. New Engl J Med.

[25] McLaughlin CC, Baptiste MS, Schymura MJ, Nasca PC, Zdeb MS (2006). Maternal and infant birth characteristics and hepatoblastoma. Am J Epidemiol.

[26] Palmer AC (2011). Nutritionally mediated programming of the developing immune system. Advances Nutr.

[27] Gopinath B, Baur LA, Wang JJ, Teber E, Liew G, Cheung N, Wong TY, Mitchell P (2010). Smaller birth size is associated with narrower retinal arterioles in early adolescence. Microcirculation.

[28] Hellstrom BE (1956). Experimental approach to the pathogenesis of retrolental fibroplasia. V. The influence of the state of nutrition on oxygen-induced changes in the mouse eye. Acta Paediatr.

[29] Orjuela MA, Cabrera-Munoz L, Paul L, Ramirez-Ortiz MA, Liu X, Chen J, Mejia-Rodriguez F, Medina-Sanson A, Diaz-Carreno S, Suen IH (2012). Risk of retinoblastoma is associated with a maternal polymorphism in dihydrofolatereductase (DHFR) and prenatal folic acid intake. Cancer.

[30] Winer RL, Hughes JP, Feng Q, O’Reilly S, Kiviat NB, Holmes KK, Koutsky LA (2006). Condom use and the risk of genital human papillomavirus infection in young women. New Engl J Med.

[31] Hogewoning CJ, Bleeker MC, van den Brule AJ, Voorhorst FJ, Snijders PJ, Berkhof J, Westenend PJ, Meijer CJ (2003). Condom use promotes regression of cervical intraepithelial neoplasia and clearance of human papillomavirus: a randomized clinical trial. Int J Cancer.

[32] Park H, Lee SW, Lee IH, Ryu HM, Cho AR, Kang YS, Hong SR, Kim SS, Seong SJ, Shin SM (2012). Rate of vertical transmission of human papillomavirus from mothers to infants: relationship between infection rate and mode of delivery. Virology J.

[33] Jones EE, Wells SI (2006). Cervical cancer and human papillomaviruses: inactivation of retinoblastoma and other tumor suppressor pathways. Curr Mol Med.

[34] Centers for Disease Control and Prevention, American Society for Reproductive Medicine, Society for Assisted Reproductive Technology (2013). 2011 Assisted reproductive technology fertility clinic success rates report.

[35] Heck JE, Lombardi CA, Cockburn M, Meyers TJ, Wilhelm M, Ritz B (2013). Epidemiology of Rhabdoid Tumors of Early Childhood. Pediatr Blood Cancer.

[36] Heck JE, Meyers TJ, Lombardi C, Park AS, Cockburn M, Reynolds P, Ritz B (2013). Case–control study of birth characteristics and the risk of hepatoblastoma. Cancer Epidemiol.

[37] Shrestha A, Ritz B, Ognjanovic S, Lombardi CA, Wilhelm M, Heck JE (2013). Early life factors and risk of childhood rhabdomyosarcoma. Frontiers in Public Health.

[38] Kallen B, Finnstrom O, Lindam A, Nilsson E, Nygren KG, Olausson PO (2010). Cancer risk in children and young adults conceived by in vitro fertilization. Pediatrics.

[39] Neelanjana M, Sabaratnam A (2008). Malignant conditions in children born after assisted reproductive technology. Obstet Gynecol Surv.

[40] LaKind JS, Amina Wilkins A, Berlin CM (2004). Environmental chemicals in human milk: a review of levels, infant exposures and health, and guidance for future research. Toxicol Appl Pharmacol.

[41] Werler MM, Mitchell AA, Hernandez-Diaz S, Honein MA (2005). Use of over-the-counter medications during pregnancy. Am J Obstet Gynecol.

[42] Rebordosa C, Kogevinas M, Bech BH, Sorensen HT, Olsen J (2009). Use of acetaminophen during pregnancy and risk of adverse pregnancy outcomes. Int J Epidemiol.

[43] Scialli AR, Ang R, Breitmeyer J, Royal MA (2010). Childhood asthma and use during pregnancy of acetaminophen. A critical review. Reprod Toxicol.

[44] Liew Z, Ritz B, Rebordosa C, Lee PC, Olsen J (2014). Acetaminophen Use During Pregnancy, Behavioral Problems, and Hyperkinetic Disorders. JAMA Pediatr.

[45] Githens PB, Glass CA, Sloan FA, Entman SS (1993). Maternal recall and medical records: an examination of events during pregnancy, childbirth, and early infancy. Birth.

[46] Olson JE, Shu XO, Ross JA, Pendergrass T, Robison LL (1997). Medical record validation of maternally reported birth characteristics and pregnancy-related events: a report from the Children’s Cancer Group. Am J Epidemiol.

[47] Feldman Y, Koren G, Mattice K, Shear H, Pellegrini E, MacLeod SM (1989). Determinants of recall and recall bias in studying drug and chemical exposure in pregnancy. Teratology.

[48] Rockenbauer M, Olsen J, Czeizel AE, Pedersen L, Sorensen HT (2001). Recall bias in a case–control surveillance system on the use of medicine during pregnancy. Epidemiology.

[49] Roberts CL, Bell JC, Ford JB, Hadfield RM, Algert CS, Morris JM (2008). The accuracy of reporting of the hypertensive disorders of pregnancy in population health data. Hypertension in Pregnancy.

[50] Lydon-Rochelle MT, Holt VL, Cardenas V, Nelson JC, Easterling TR, Gardella C, Callaghan WM (2005). The reporting of pre-existing maternal medical conditions and complications of pregnancy on birth certificates and in hospital discharge data. Am J Obstet Gynecol.

[51] Lawrence JM, Contreras R, Chen W, Sacks DA (2008). Trends in the prevalence of preexisting diabetes and gestational diabetes mellitus among a racially/ethnically diverse population of pregnant women, 1999–2005. Diabetes Care.

[52] Kwon HL, Belanger K, Bracken MB (2003). Asthma prevalence among pregnant and childbearing-aged women in the United States: estimates from national health surveys. Ann Epidemiol.

[53] Casey BM, Dashe JS, Wells CE, McIntire DD, Byrd W, Leveno KJ, Cunningham FG (2005). Subclinical hypothyroidism and pregnancy outcomes. Obstet Gynecol.

[54] Le Strat Y, Dubertret C, Le Foll B (2011). Prevalence and correlates of major depressive episode in pregnant and postpartum women in the United States. J Affect Disord.

[55] Melville JL, Gavin A, Guo Y, Fan MY, Katon WJ (2010). Depressive disorders during pregnancy: prevalence and risk factors in a large urban sample. Obstet Gynecol.

