# Supplementary material for: A case–control study of sporadic retinoblastoma in relation to maternal health conditions and reproductive factors: a report from the Children’s Oncology group
Source: BMC Cancer. 2015 Oct 19;15:735. doi: 10.1186/s12885-015-1773-0 (PMC4615328; doi:10.1186/s12885-015-1773-0)
Supplement: Additional file 1: — Table S1. Multiple imputation/propensity score analysis of retinoblastoma in relation to maternal medical conditions and prescription drug use which occurred in the month before or during the pregnancy. Table S2. Multiple imputation/propensity score analysis of associations between maternal pregnancy history, body size, and breastfeeding with retinoblastoma using unconditional logistic regression. Table S3. Multiple imputation/propensity score analysis of sporadic retinoblastoma in relation to the mother’s birth control use and fertility treatment. (DOC 141 kb) [file 12885_2015_1773_MOESM1_ESM.doc]

**Table S1. Multiple imputation/propensity score analysis of r**etinoblastoma in relation to maternal medical conditions and prescription drug use which occurred in the month before or during the pregnancy

|  | **Controls**  **(n=146)** | **Unilateral cases (n=185)** | | |  | **Bilateral cases (n=95)** | | |
| --- | --- | --- | --- | --- | --- | --- | --- | --- |
|  |  |  | **Crude^a^** | **Adjusted^b^** |  |  | **Crude^a^** | **Adjusted^b^** |
| ***Medical conditions*** | N (%) | N (%) | OR | OR (95% CI) |  | N (%) | OR | OR (95% CI) |
| **Any medical condition** | 74 (47.7) | 115 (61.5) | 1.6 | 1.5 (1.0, 2.3) |  | 57 (60.0) | 1.2 | 1.1 (0.6, 1.9) |
| **Infectious diseases** |  |  |  |  |  |  |  |  |
| Any infectious disease | 32 (20.7) | 44 (23.6) | 1.1 | 1.1 (0.6, 1.8) |  | 19 (20.0) | 0.8 | 0.7 (0.3, 1.3) |
| Respiratory infection | 18 (11.6) | 21 (11.2) | 1.0 | 1.0 (0.5, 2.0) |  | 7 (7.4) | 0.5 | 0.4 (0.1, 1.2) |
| Flu or cold | 10 (6.5) | 17 (9.1) | 1.4 | 1.3 (0.6, 3.0) |  | 3 (3.4) | 0.4 | 0.3 (0.1, 1.4) |
| Other viral infections | 1 (0.7) | 5 (2.7) | 3.8 | 7.1 (0.6, 80.8) |  | 2 (2.1) | 2.2 | 3.4 (0.3, 38.4) |
| Sexually transmitted infections | 0 (0.0) | 1 (0.5) | - | - |  | 1 (1.1) | - | - |
| All bacterial infections | 18 (11.6) | 24 (12.8) | 1.1 | 1.1 (0.6, 2.1) |  | 11 (11.6) | 0.9 | 0.7 (0.3, 1.7) |
| Urinary tract infections | 6 (3.9) | 9 (4.8) | 1.1 | 1.0 (0.3, 3.0) |  | 1 (1.1) | 0.1 | 0.1 (0.0, 1.3) |
| Mother took antibiotics in pregnancy | 21 (13.6) | 22 (11.8) | 0.8 | 0.9 (0.4, 1.6) |  | 10 (10.5) | 0.6 | 0.6 (0.3, 1.4) |
| **Chronic diseases** |  |  |  |  |  |  |  |  |
| High blood pressure | 17 (11.0) | 23 (12.3) | 1.1 | 1.2 (0.6, 2.3) |  | 11 (11.6) | 1.2 | 0.9 (0.4, 2.0) |
| Gestational high blood pressure | 17 (11.0) | 22 (11.8) | 1.1 | 1.1 (0.6, 2.2) |  | 11 (11.6) | 1.2 | 0.9 (0.4, 2.0) |
| Diabetes (any) | 6 (3.9) | 17 (9.1) | 2.5 | 2.3 (0.9, 6.0) |  | 8 (8.4) | 1.8 | 2.1 (0.7, 6.2) |
| Gestational diabetes | 6 (3.9) | 14 (7.5) | 2.0 | 1.9 (0.7, 5.1) |  | 8 (8.4) | 1.8 | 2.0 (0.7, 6.1) |
| Preeclampsia | 7 (4.5) | 9 (4.8) | 1.1 | 1.0 (0.4, 2.8) |  | 2 (2.1) | 0.7 | 0.4 (0.1, 2.2) |
| **Endocrine disorders** |  |  |  |  |  |  |  |  |
| Any thyroid problem | 2 (1.3) | 6 (3.2) | 2.2 | 2.0 (0.4, 10.5) |  | 2 (2.1) | 1.0 | 1.0 (0.1, 7.7) |
| Hypothyroidism | 2 (1.3) | 5 (2.7) | 1.6 | 1.6 (0.3, 9.6) |  | 2 (2.1) | 1.0 | 0.8 (0.1, 7.4) |
| **Pain and pain medication** |  |  |  |  |  |  |  |  |
| Back pain | 4 (2.6) | 11 (5.9) | 2.5 | 2.7 (0.8, 9.0) |  | 4 (4.2) | 2.2 | 1.6 (0.4, 7.2) |
| Pain medication | 3 (1.9) | 8 (4.3) | 2.3 | 4.0 (0.8, 16.3) |  | 7 (7.4) | 6.0 | 3.7 (0.9, 16.1) |
| **Other Diseases and conditions** |  |  |  |  |  |  |  |  |
| Depression/anxiety | 5 (3.2) | 10 (5.4) | 2.2 | 2.1 (0.6, 7.2) |  | 5 (5.3) | 2.3 | 2.7 (0.6, 11.7) |
| Anemia | 1 (0.7) | 6 (3.2) | 4.5 | 3.3 (0.4, 29.9) |  | 2 (2.1) | 2.2 | 2.3 (0.2, 27.1) |
| Allergy/asthma | 8 (5.2) | 13 (7.0) | 1.3 | 1.3 (0.5, 3.3) |  | 7 (7.4) | 1.3 | 1.2 (0.4, 3.5) |

^a^Adjusted for the matching variable, child’s age at interview.

^b^Adjusted for child age at interview, mother’s race/ethnicity, mother’s educational attainment, household income, mother’s age at birth, and maternal smoking in the month before or during pregnancy.

**Table S2. Multiple imputation/propensity score analysis of a**ssociations between maternal pregnancy history, body size, and breastfeeding with retinoblastoma using unconditional logistic regression

|  | **Controls** | **Unilateral cases (n=185)** | | |  | **Bilateral cases (n=95)** | | |
| --- | --- | --- | --- | --- | --- | --- | --- | --- |
|  | (N=146) |  | **Crude^a^** | **Adjusted^b^** |  |  | **Crude^a^** | **Adjusted^b^** |
|  | N (%) | N (%) | OR | OR (95% CI) |  | N (%) | OR | OR (95% CI) |
| **Maternal history of stillbirth** | 1 (0.7) | 5 (2.7) | 4.7 | 4.2 (0.2, 88.0) |  | 2 (2.1) | 4.1 | 2.4 (0.0,120.9) |
| **Order of index pregnancy** |  |  |  |  |  |  |  |  |
| First pregnancy | 55 (38.2) | 60 (32.6) | 1.0 | Referent |  | 33 (35.1) | 1.0 | Referent |
| Second pregnancy | 39 (27.1) | 57 (31.0) | 1.4 | 1.3 (0.7, 2.2) |  | 32 (34.0) | 1.6 | 1.5 (0.8, 2.9) |
| Third and above | 50 (34.7) | 67 (36.4) | 1.3 | 0.9 (0.5, 1.7) |  | 29 (30.9) | 1.1 | 0.8 (0.4, 1.7) |
| **Order of index child** |  |  |  |  |  |  |  |  |
| First child | 71 (49.3) | 86 (46.7) | 1.0 | Referent |  | 46 (49.5) | 1.0 | Referent |
| Second child | 40 (27.8) | 68 (37.0) | 1.5 | 1.5 (0.9, 2.5) |  | 27 (29.0) | 1.2 | 1.3 (0.7, 2.6) |
| Third and above | 33 (22.9) | 30 (16.3) | 0.8 | 0.6 (0.3, 1.2) |  | 20 (21.5) | 1.1 | 0.8 (0.4, 1.8) |
| **Ever breastfed index child** | 120 (85.1) | 137 (74.9) | 0.5 | 0.6 (0.3, 1.1) |  | 74 (79.6) | 0.6 | 0.7 (0.3, 1.5) |
| **Breastfeeding duration** |  |  |  |  |  |  |  |  |
| 0 month | 25 (18.7) | 47 (28.5) | 1.0 | Referent |  | 19 (24.4) | 1.0 | Referent |
| 1-6 months | 66 (49.3) | 67 (40.6) | 0.5 | 0.6 (0.3, 1.1) |  | 36 (46.2) | 0.7 | 0.8 (0.4, 1.7) |
| 7-11 months | 21 (15.7) | 16 (9.7) | 0.4 | 0.5 (0.2, 1.2) |  | 7 (9.0) | 0.4 | 0.5 (0.2, 1.4) |
| 12+ months | 22 (16.4) | 35 (21.2) | 0.8 | 1.2 (0.6, 2.6) |  | 16 (20.5) | 1.1 | 1.4 (0.5, 3.7) |
| **Gave index child formula while breastfeeding** | 68 (58.1) | 78 (56.9) | 1.0 | 0.9 (0.5, 1.5) |  | 38 (54.3) | 0.8 | 0.7 (0.4, 1.4) |
| **Mother’s BMI at the start of pregnancy** |  |  |  |  |  |  |  |  |
| Underweight (BMI:<18.5) | 5 (3.5) | 10 (5.4) | 1.9 | 1.8 (0.5, 5.8) |  | 8 (8.4) | 3.8 | 2.3 (0.7, 7.7) |
| Normal (BMI: 18.5-<25) | 90 (62.1) | 101 (54.9) | 1.0 | Referent |  | 50 (52.6) | 1.0 | Referent |
| Overweight (BMI:25-<30) | 31 (21.4) | 45 (24.5) | 1.3 | 1.2 (0.7, 2.1) |  | 20 (21.1) | 1.3 | 1.0 (0.5, 2.0) |
| Obese (BMI: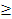30) | 19 (13.1) | 28 (15.2) | 1.4 | 1.0 (0.5, 2.1) |  | 17 (17.9) | 1.6 | 1.5 (0.7, 3.2) |
| **Weight gain in pregnancy** |  |  |  |  |  |  |  |  |
| Low weight gain | 23 (17.0) | 26 (14.7) | 0.9 | 1.1 (0.5, 2.3) |  | 12 (13.0) | 0.8 | 1.0 (0.4, 2.4) |
| Normal weight gain | 51 (37.8) | 64 (36.2) | 1.0 | Referent |  | 34 (37.0) | 1.0 | Referent |
| High weight gain | 61 (45.2) | 87 (49.2) | 1.1 | 1.1 (0.6, 1.8) |  | 46 (50.0) | 1.0 | 0.9 (0.5, 1.7) |
| **Father’s BMI at the start of pregnancy** |  |  |  |  |  |  |  |  |
| Underweight (BMI:<18.5) | 1 (0.7) | 2 (1.2) | 2.8 | 2.6 (0.2, 32.1) |  | 2 (2.2) | 3.2 | 1.7 (0.1, 23.3) |
| Normal (BMI: 18.5-<25) | 36 (25.7) | 33 (20.1) | 1.0 | Referent |  | 32 (35.2) | 1.0 | Referent |
| Overweight (BMI:25-<30) | 57 (40.7) | 84 (51.2) | 1.6 | 1.7 (0.9, 3.0) |  | 33 (36.3) | 0.6 | 0.7 (0.4, 1.3) |
| Obese (BMI: :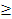30) | 76 (32.8) | 45 (27.4) | 1.1 | 1.0 (0.5, 1.8) |  | 24 (25.4) | 0.6 | 0.6 (0.3, 1.3) |

^a^ Adjusted for the matching variable, child’s age at interview.

^b^Adjusted for child age at interview, mother’s race/ethnicity, mother’s educational attainment, household income, mother’s age at birth, and maternal smoking in the month before or during pregnancy.

**Table S3. Multiple imputation/propensity score analysis of s**poradic retinoblastoma in relation to the mother’s birth control use and fertility treatment

|  | **Controls** | **Unilateral (n=185)** | | |  | **Bilateral (n=95)** | | |
| --- | --- | --- | --- | --- | --- | --- | --- | --- |
|  | (N=146) |  | **Crude^a^** | **Adjusted** |  |  | **Crude^a^** | **Adjusted** |
|  | N (%) | N (%) | OR | OR (95% CI) |  | N (%) | OR | OR (95% CI) |
| **Birth control used in the year before the index pregnancy ^b^** |  |  |  |  |  |  |  |  |
| Any birth control | 75 (52.5) | 85 (46.6) | 0.8 | 0.9 (0.6, 1.4) |  | 45 (47.9) | 0.8 | 1.0 (0.6, 1.7) |
| Oral contraceptive pills | 43 (29.3) | 60 (32.4) | 1.2 | 1.4 (0.9, 2.3) |  | 28 (29.5) | 0.8 | 1.2 (0.7, 2.2) |
| Injection, Implant, skin patch, Vaginal ring | 9 (6.1) | 13 (7.0) | 1.2 | 0.9 (0.3, 2.2) |  | 7 (7.4) | 1.0 | 1.0 (0.3, 3.0) |
| Any hormonal contraceptive | 49 (33.3) | 71 (38.4) | 1.3 | 1.4 (0.9, 2.3) |  | 34 (35.8) | 0.9 | 1.3 (0.7, 2.2) |
| Condoms | 26 (17.7) | 15 (8.1) | 0.4 | 0.5 (0.3, 1.1) |  | 14 (14.7) | 0.9 | 1.0 (0.5, 2.1) |
| Intra uterine device (IUD) | 4 (2.7) | 3 (1.6) | 0.6 | 0.3 (0.1, 1.7) |  | 1 (1.1) | 0.4 | 0.5 (0.1, 5.9) |
| Other type of birth control | 2 (1.4) | 2 (1.1) | 0.9 | 1.9 (0.2, 16.0) |  | 0 (0.0) | - | - |
| **Child conceived with the help of fertility treatment ^c^** |  |  |  |  |  |  |  |  |
| Any type of fertility treatment used by either parent | 9 (6.3) | 14 (7.7) | 1.1 | 1.6 (0.6, 4.3) |  | 8 (8.5) | 1.2 | 1.1 (0.4, 3.4) |
| Mother took fertility medication for this pregnancy | 8 (5.6) | 14 (7.6) | 1.3 | 1.7 (0.6, 4.6) |  | 6 (6.4) | 1.0 | 1.1 (0.4, 3.6) |
| Used IVF for this pregnancy | 0 (0) | 7 (3.8) | - | - |  | 5 (5.3) | - | - |
| Used artificial intrauterine insemination | 2 (1.4) | 3 (1.6) | 1.1 | 1.5 (0.2, 9.3) |  | 3 (3.2) | 2.0 | 1.4 (0.2, 10.0) |
| Used Intra Cytoplasmic Sperm Injection (ICSI) | 1 (0.7) | 3 (1.6) | 1.9 | 4.7 (0.3,71.4) |  | 2 (2.1) | 1.9 | 3.1 (0.2, 40.9) |
| Problem with egg was the reason for infertility treatment | 2 (1.4) | 2 (1.1) | 0.9 | 1.2 (0.1, 10.0) |  | 4 (4.3) | 2.9 | 5.1 (0.8, 34.0) |
| Problem with sperm was the reason for infertility treatment | 0 (0.0) | 3 (1.6) | - | - |  | 2 (2.1) | - | - |
| The reason for infertility treatment was unknown | 3 (2.1) | 7 (3.8) | 1.6 | 2.7 (0.6, 13.1) |  | 2 (2.1) | 0.8 | 0.9 (0.1, 6.1) |

^a^ Adjusted for matching variable, child’s age at interview.

^b^Adjusted for child age at interview, mother’s race/ethnicity, mother’s educational attainment, household income, and maternal smoking in the month before or during pregnancy.

**^c^** Adjusted for child’s age at interview, mother’s race/ethnicity, mother’s educational attainment and household income
